# The Effects of Attentional Engagement on Route Learning Performance in a Virtual Environment: An Aging Study

**DOI:** 10.3389/fnagi.2017.00235

**Published:** 2017-07-20

**Authors:** Steffen Hartmeyer, Ramona Grzeschik, Thomas Wolbers, Jan M. Wiener

**Affiliations:** ^1^Department of Psychology, Bournemouth University Poole, United Kingdom; ^2^Ageing and Dementia Research Centre, Bournemouth University Poole, United Kingdom; ^3^German Centre for Neurodegenerative Diseases Magdeburg, Germany

**Keywords:** aging, navigation, attention, route learning, attentional engagement, decision points, auditory probe task, selective attention

## Abstract

Route learning is a common navigation task affected by cognitive aging. Here we present a novel experimental paradigm to investigate whether age-related declines in executive control of attention contributes to route learning deficits. A young and an older participant group was repeatedly presented with a route through a virtual maze comprised of 12 decision points (DP) and non-decision points (non-DP). To investigate attentional engagement with the route learning task, participants had to respond to auditory probes at both DP and non-DP. Route knowledge was assessed by showing participants screenshots or landmarks from DPs and non-DPs and asking them to indicate the movement direction required to continue the route. Results demonstrate better performance for DPs than for non-DPs and slower responses to auditory probes at DPs compared to non-DPs. As expected we found slower route learning and slower responses to the auditory probes in the older participant group. Interestingly, differences in response times to the auditory probes between DPs and non-DPs can predict the success of route learning in both age groups and may explain slower knowledge acquisition in the older participant group.

## Introduction

During navigating we are exposed to a multitude of information, both visual and non-visual, only some of which is navigationally relevant. Moreover, in real world navigation we are typically engaged in other tasks as well, such as, monitoring traffic. Successful and safe navigation therefore requires focusing attention on the actual navigation task when approaching navigationally relevant situations and freeing up attentional resources for other tasks when not required for navigation (i.e., disengaging with the navigation task). In this study we use an auditory-probe task to (1) study how attentional engagement with the route learning task in a virtual environment is modulated depending on the relevance of the situation and (2) whether age-related route learning deficits can, at least partly, be explained by less effective attentional engagement.

Route navigation—following a known path from an origin to a destination—is arguably the most frequent human navigation tasks. While route navigation can be supported by different strategies (see Waller and Lippa, 2007), navigators typically need to memorize a series of movement directions at decision points to successfully navigate a route. The most parsimonious form of route knowledge—which is only suited for relatively short routes or in situation in which landmarks are unavailable —is simply a series of direction changes (such as, “left, right, right, straight”; cf. Waller and Lippa, 2007). If the environment features landmarks, however, navigators tend to use these for stimulus-response strategies where an action—such as, a turn—is triggered by the recognition of a landmark (Trullier et al., 1997). Depending on their positioning, landmarks can serve as beacons or associative cues. If positioned such that (i) landmarks are visible from a decision point and (ii) that approaching them brings the navigator closer to the destination of the route, they can serve as beacons (“Walk toward the church”). The more commonly reported route learning strategy, however, is the associative-cue strategy where landmarks serve as cues for actions (Siegel and White, 1975; O'Keefe and Nadel, 1978). Here, landmarks become associated with motor responses that are related to the navigator's body axis (“Turn left at the church,” Wolbers and Wiener, 2014). In contrast to the beacon strategy, the associative cue strategy requires the explicit encoding of directional information.

While landmark-based route learning strategies are more efficient than simply memorizing a series of direction changes (Waller and Lippa, 2007), not all environmental cues or objects along a route are equally relevant for navigation and make good landmarks. For successful route navigation, navigators need to encode and remember a number of direction changes which usually take place at decision points. Consequently, objects located at decision points are remembered faster and more reliably than those located at non-decision points (straight segment or simple turn; Aginsky et al., 1997; Janzen, 2006) and result in increased parahippocampal gyrus activation (Janzen and van Turennout, 2004; Janzen and Weststeijn, 2007; Schinazi and Epstein, 2010). These results demonstrate that navigators, when learning unfamiliar routes, pay particular attention to situations that require navigational decisions, while fewer attentional resources are devoted to monitor other parts of the route.

Such allocation of attentional resources to focus on a subset of relevant information while ignoring irrelevant distractors is particularly important if the task demands are high and perceptual capacities are exceeded (Zanto and Gazzaley, 2014). The allocation of cognitive resources to the main task decreases the likelihood that critical aspects go unnoticed, supports the suppression of task irrelevant information and facilitates successful encoding of selected information (Lavie and Dalton, 2014). The degree of attentional resources and cognitive effort engaged with a task can be assessed by examining the response time to a secondary task. Generally, the more attention is engaged with a primary task (e.g., watching television), the longer it takes to disengage from that task and to respond to a secondary task, such as, pressing a button in response to a tone (Basil, 1994). This is, because fewer cognitive resources are available for the processing of other incoming stimuli.

So far, only few studies have explicitly studied attentional engagement during route learning, i.e., the degree to which effort is directed to the actual navigation task. Allen and Kirasic (2003) presented participants with a slide-presentation simulation of a route featuring high and low information regions. High and low information regions along the route were identified by independent raters on the basis of how useful these were to a navigator for knowing where they were along the route and how to get to the end of the walk. Unfortunately, little further information about the actual content of high/low information slides was given. During encoding of the route, attentional engagement with the route learning task was monitored using an auditory-probe task. Specifically, participants were asked to respond to an occasional auditory stimulus as quickly as possible. Results demonstrated that participants were slower to respond to the auditory probe when inspecting slides depicting high information regions as compared to low information regions. Here we develop Allen and Kirasic's paradigm further and use it to investigate whether control of attentional engagement contributes to the route-learning deficits reported in healthy aging older adults.

Several studies have now described route learning deficits in older adults (e.g., Barrash, 1994; Wilkniss et al., 1997; Moffat et al., 2001; Moffat, 2009; Head and Isom, 2010; Wiener et al., 2012; Merriman et al., 2016; Zhong and Moffat, 2016). Route navigation depends on a number of processes which are affected by aging. For example, older adults, after being exposed to an unfamiliar route as often as younger adults, or for the same amount of time, show less accurate knowledge of the direction in which the route continues at particular landmarks or intersections (Head and Isom, 2010; Liu et al., 2011; Wiener et al., 2012; Zhong and Moffat, 2016) and less accurate knowledge of the sequence of landmarks (Wilkniss et al., 1997; Head and Isom, 2010; Wiener et al., 2012; Merriman et al., 2016). The ability to freely recall or recognize landmarks that were encountered along the route, in contrast, often remains unaffected (Cushman et al., 2008; Head and Isom, 2010; Zhong and Moffat, 2016). While neurodegeneration of the caudate nucleus has been associated with aging related declines in route learning performance (Head and Isom, 2010), the exact mechanism of this decline remains unclear. One finding in particular, however, suggests that route learning deficits may result from attentional processes. Lipman (1991) reported that older adults are more likely to point out salient landmarks than turns as route critical elements. This suggests that older adults may evaluate the navigational relevance of spatial situations differently to younger participants. This could lead to attentional disengagement or reduced attentional engagement with navigationally relevant situations which, in turn, could contribute to the observed aging-related route learning deficits. This is in line also with research in other cognitive domains which demonstrated that older adults are more easily distracted by task-irrelevant stimuli due to age-related deficits in attentional regulation for the suppression of task irrelevant stimuli (Lavie et al., 2004; Gazzaley et al., 2005; Zanto et al., 2010). Generally, such age-related declines in selective attention might be related to perceptual and cognitive load capacity limitations (Zanto and Gazzaley, 2014).

In this study we present a route learning paradigm inspired by Allen and Kirasic (2003). Specifically, we presented participants with a video of a long route through a virtual environment that consists of decision points, straight segments and simple turns, thus systematically manipulating the navigational relevance of spatial situations along the route. We used an auditory-probe task to measure attentional engagement during the learning of the route (cf. Allen and Kirasic, 2003). To investigate whether attentional engagement contributes to aging-related route-learning deficits we compared route-learning performance and response times to the auditory probe in different spatial situations between a young and an older participant group.

Following on from earlier research (Allen and Kirasic, 2003), we expected longer response times to the auditory probe at decision points (DP) as compared to non-decision points (non-DP). Moreover, if the auditory-probe task actually captured attentional engagement that was relevant for navigational success, we expected that larger differences in response times between DP and non-DP correlated with route learning performance. Based on previous studies, we also expected better performance at DP as compared to non-DP (Schinazi and Epstein, 2010; Kessels et al., 2011). With respect to aging, we expected slower route learning and generally slower response times to the auditory probe in our older participant group than in the younger group. If control of attentional engagement was affected by aging, we expected reduced differences in response time to auditory probe between DP and non-DP in older participants and a weaker correlation between these differences in response times and route learning performance.

## Materials and methods

The design of the current experiment was inspired by Allen and Kirasic (2003). However, rather than presenting a slideshow of pictures taken along a route, we used a walk through a virtual maze. This allowed for improved control of environmental cues and spatial features. Also, instead of probing route knowledge by asking participants to judge inter-location distances (Allen and Kirasic, 2003), we used a more traditional measure of route knowledge, i.e., we presented participants with pictures of landmarks or spatial situations and asked them to indicate the direction in which the route continued (Wiener et al., 2013; Strickrodt et al., 2015; de Condappa and Wiener, 2016).

### Participants

Forty six participants [23 young adults (12 females; mean age 20.57 ± 2.57 years; range, 18–28) and 23 older adults (11 females; mean age 72.17 ± 5.56 years; range, 63–85)] took part in the experiment. As this study was not designed to investigate potential gender differences and as exploratory analyses did not reveal any gender effects, we did not include gender as a factor in the further analysis. We administered the Montreal Cognitive Assessment (MoCA; Nasreddine et al., 2005) to the older participants to screen for mild cognitive impairment. One older participant was excluded based on the recently recommended MoCA cutoff score for MCI of 23 (Luis et al., 2009). The remaining older participants had a mean MoCa score 26.74 (range 23–30). Most of the younger participants were Psychology undergraduates at Bournemouth University who and were rewarded course credits for their participation. The older participants were volunteers who were reimbursement for their participation in the study. Ethical approval was obtained from the Science, Technology and Health Research Ethics Panel at Bournemouth University and written informed consent was obtained from all participants, in accordance with the declaration of Helsinki.

### Virtual environment

**Figure 1:**
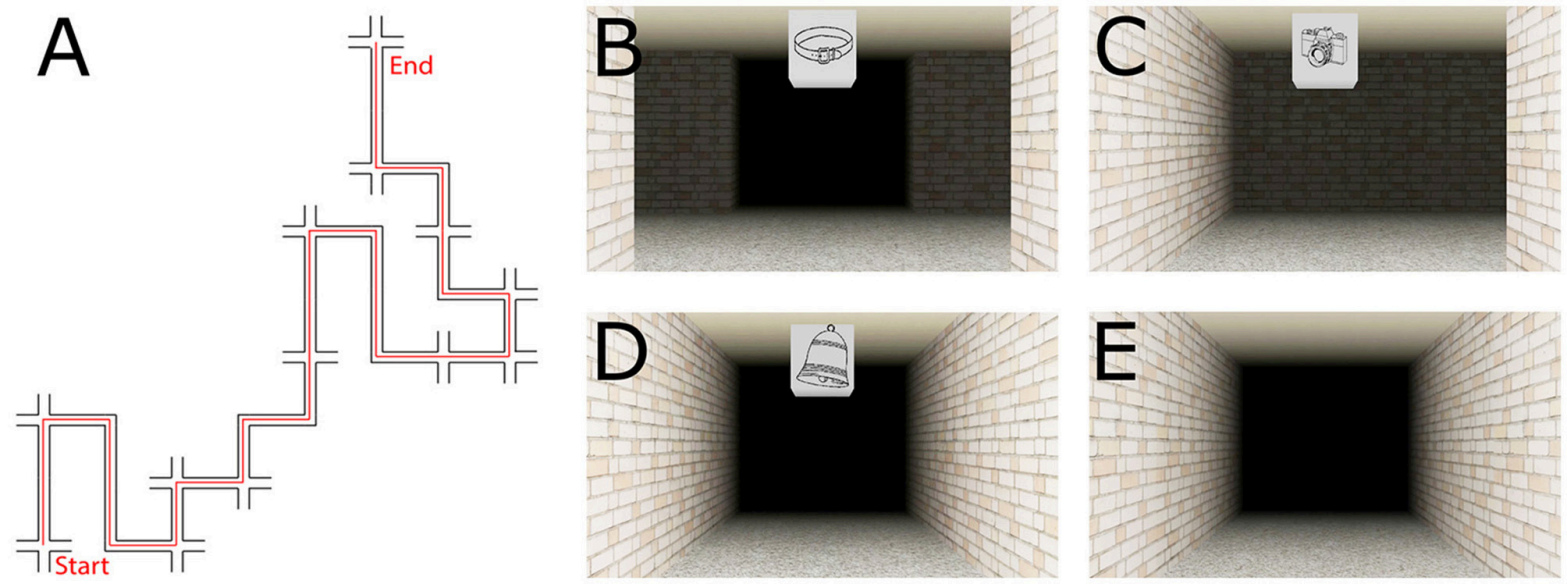
Schematic of the route **(A)** and exemplary pictures of the intersection types: decision point **(B)**, non-decision point turn **(C)**, non-decision point straight **(D)**, corridor without landmark **(E)**. The experiment consisted of three distinct phases.

The virtual route was created using Vizard 3.0 (WorldViz, Santa Barbara, USA). The route comprised 12 decision points (DP, i.e., four-way intersections, see Figure 1B), and 12 non-decision points (non-DP; i.e., straight segments or turns with only one possible movement direction, see Figures 1C,D). For both DPs as well as for non-DPs there were three left turns, three right turns, and three straight movements distributed along the route (see Figure 1A). Each DP and non-DP featured a unique landmark object, a line drawing of an object that was mapped onto a cube suspended from the ceiling (see Figures 1B–D). The landmark objects were selected to contain as little directional information as possible. We introduced black fog in the environment to ensure that only one landmark and section/intersection was visible at any time. The video is available in the Supplementary Material.

### Training phase and auditory-probe task

During the training phase, participants were passively transported along the route at walking speed (2 ms with a camera height of 1.60 m). The walkthrough was presented on a 22″ LCD monitor (resolution 1,920 × 1,080 pixels, refresh rate 60 Hz). Participants were instructed to learn the route so that they would be able to repeat it on their own. During the route presentation, an auditory stimulus (100 ms, square wave 1,000 Hz) was repeatedly presented via headphones using an external sound card (ASIO M-Track Plus, M-Audio, Cumberland, USA). Participants were instructed to respond to the stimulus as fast as possible by pressing a key on a response box (RB-740, Cedrus, San Pedro, USA). Eighteen auditory stimuli were distributed over the entire route. Six auditory probes were presented at DPs, six auditory probes were presented at non-DPs, and six auditory probes were presented between DPs or non-DPs (i.e., along a corridor without a landmark, see Figure 1E). To further reduce the predictability of when an auditory stimulus was presented, they were presented either exactly under a landmark (0 ms) or 667 ms before the landmark was reached. These presentation times were counterbalanced between location type (DP, non-DP) and movement direction (left, right, straight). The first key press within 2,000 ms after an auditory stimulus was presented was recorded. The training phase was designed to test participants' engagement with the route learning task. More specifically, we were interested in changes in attentional engagement depending on the relevance of the spatial situation.

### Landmark-in-context test

In the Landmark-In-Context test, participants were presented with screenshots of all decision points and all non-decision points in randomized order. Their task was to indicate the movement direction required to follow the route by pressing the corresponding button on the response box. Note that the required movement direction for non-decision points, i.e., a simple turn or straight corridor, was apparent in the screenshot and therefore did not have to be learned during the route presentation. The Landmark-In-Context test allowed us to monitor route learning over the course of the experiment.

### Landmark-only test

The task was similar to the Landmark-In-Context test but instead of showing screenshots, we presented participants with isolated images of the landmark objects at decision and non-decision points in randomized order. Again, participants' task was to indicate the movement direction in which the route continued. In contrast to the Landmark-In-Context test, the movement direction required at non-DP was not coded in the stimulus itself but had to be retrieved from memory. Participants were not initially informed of this test and only received instructions directly before the test was administered. The Landmark-Only test is similar to the Landmark-In-Context test but allowed us to compare landmark-movement associations between decision points and non-decision points.

### Procedure

Before the actual experiment, participants received a short practice session to familiarize them with the tasks. To do so, we created a short route that was different to the actual experimental route and contained different landmarks. In the practice session we presented participants with one training phase (including the auditory probe task) and one Landmark-In-Context phase. As the Landmark-Only test was designed as a surprise task it was not used in the practice session.

The experiment consisted of three sessions. Sessions 1 and 2 were comprised of a *Training Phase* and a subsequent *Landmark-In-Context* test. Session 3 was comprised of a *Training Phase* and a subsequent *Landmark-Only* test.

## Results

### Landmark-in-context test

**Figure 2:**
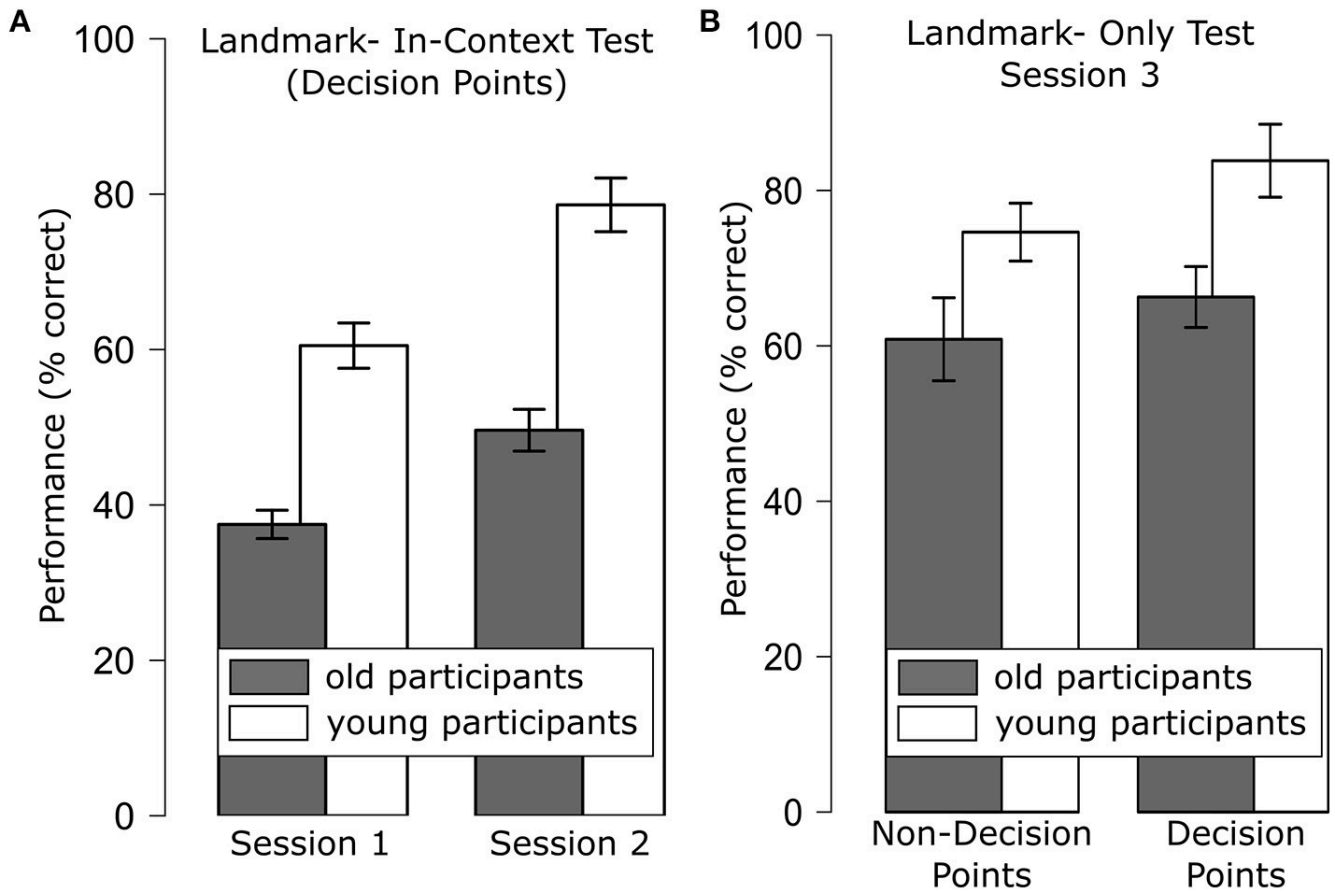
**(A)** Route learning performance for decision points for the Landmark-in-Context test that was administered after the first and second training session which rendered significant main effects of age group and session; **(B)** performance for the Landmark-Only test that was administered after the third training session for landmarks at decision points and landmarks at non-decision points, which rendered significant main effects of age group and decision point. Error bars are standard error of the mean.

We only analyzed responses of decision points as the movement direction at non-decision points was coded in the actual stimulus. A repeated measures ANOVA with the between-subjects factor age group (young, old) and the within-subjects factors session (1–2) and movement direction (straight, turn) revealed main effects of age group [*F*_(1, 43)_ = 24.53, *p* < 0.001], session [*F*_(1, 43)_ = 27.35, *p* < 0.001] and movement direction [*F*_(1, 43)_ = 14.50, *p* < 0.001]. Specifically, performance for decision points was better for the young than for the old participant group (young: 69.57%; old: 43.56%), performance improved over sessions (session 1: 49.26%; session 2: 64.44%) and performance was better for straight movements (66.39%) than for turns (52.08%). Note that performance in the older participants in session 1 did not differ significantly from chance level [37.50 vs. 33.33%; *t*_(21)_ = 1.56; *p* = 0.13], while performance in session 2 and performance in the younger participants in both session 1 and session 2 clearly exceeded chance level (all *p* < = 0.001, see Figure 2A). None of the interactions were significant (all *p* > 0.05).

### Landmark-only test

An ANOVA with the between factor age group (young, old) and the within factors decision (DP, non-DP) and movement direction (straight, turn) revealed main effects of age group [*F*_(1, 43)_ = 9.77, *p* < 0.01, see Figure 2B], decision point [*F*_(1, 43)_ = 7.51, *p* < 0.01] as well as of movement direction [*F*_(1, 43)_ = 10.79, *p* < 0.01]. Specifically, younger participants performed better than older participants (79.25 vs. 63.59%), performance for decision points was better than for non-decision points (75.25 vs. 67.90%), and performance for straight movements was better than for turns (78.02 vs. 68.37%). None of the two-way interactions reached significance.

### Auditory-probe task

A repeated measures ANOVA with the between factor age group (young, old) and the within factors decision point (DP, non-DP), session (1–3) and movement direction (straight, turn) revealed main effects of age group [*F*_(1, 43)_ = 22.48, *p* < 0.001], decision point [*F*_(1, 43)_ = 28.57, *p* < 0.001] and movement direction [*F*_(1, 43)_ = 18.56, *p* < 0.001], but no main effect of session [*F*_(2, 86)_ = 1.71, *p* = 0.17]. Specifically, younger participants responded faster to the auditory probes than older participants (417 vs. 653 ms) and responses were faster at non-decision points than at decision points (491 vs. 574 ms).

**Figure 3:**
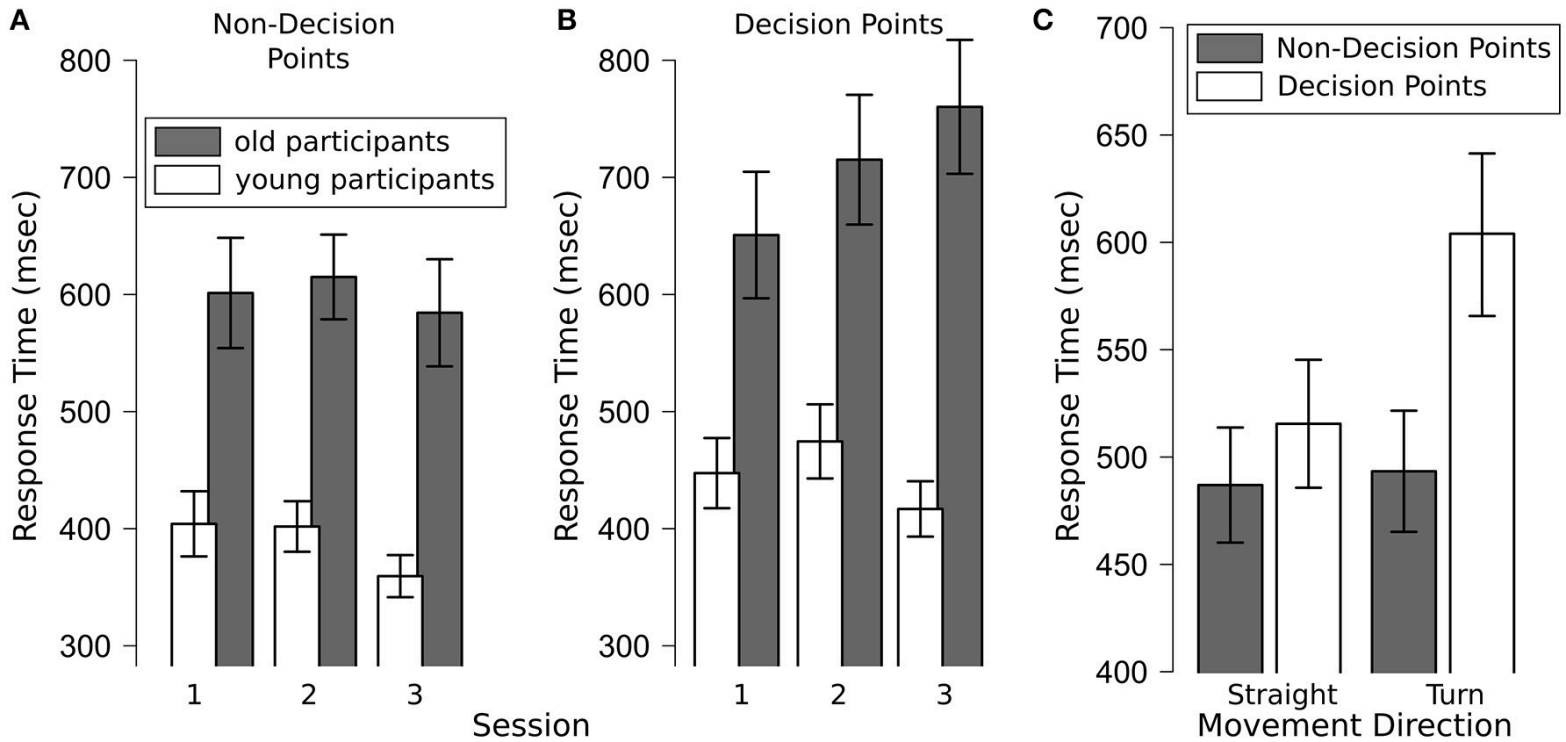
**(A)** Response times of older and younger participants to the auditory probe task at non-decision points by session which rendered significant main effects of age group and session; **(B)** response times of older and younger participants to the auditory probe task at decision points by session, which revealed a significant main effect of age group and an interaction between age group and session (*p* = 0.001); **(C)** response times to the auditory probe task for straight movements and turns at decision- and non-decision points, which revealed significant main effects of decision point and movement direction as well as a significant interaction between both factors (*p* < 0.01). Error bars are standard error of the mean.

Of the interactions, age group x movement direction [*F*_(1, 43)_ = 6.34, *p* = 0.02], age group x session [*F*_(2, 86)_ = 5.38, *p* < 0.01], decision point × movement direction [*F*_(1, 43)_ = 11.72, *p* < 0.01; see Figure 3C], decision × session [*F*_(2, 86)_ = 3.88, *p* = 0.03], and decision × movement direction × session [*F*_(2, 86)_ = 13.68, *p* < 0.001] rendered significant results. Figures of the interactions are available in the Supplementary Material.

To investigate the nature of these interactions and the nature of the three way interaction in particular, we analyzed data for decision points and non-decision points independently.

### Non-decision points

A repeated measures ANOVA with the between factor age group (young, old) and the within factors session (1–3) and movement direction (straight, turn) revealed main effects of age group [*F*_(1, 43)_ = 21.73, *p* < 0.001] and session [*F*_(2, 86)_ = 3.54, *p* = 0.03], but no effect of movement direction [*F*_(1, 43)_ = 0.04, *p* = 0.83]. Specifically, younger participants responded faster than older participants (388 vs. 598 ms) and response times increased from session 1 to session 2 and then fell in session 3 (session 1: 439 ms; session 2: 493 ms; session 3: 398 ms; see Figure 3A). *Post-hoc* analyses show that the difference between session 2 and 3 was significant.

Only the session x movement direction interaction rendered a significant result [*F*_(2, 86)_ = 6.25, *p* = 0.003; see Supplementary Figure 1C]. Further analyses of this interaction did not reveal a clear pattern to explain the interaction. Specifically, response times for turns was higher than for straight movements in session 1 (516.8 vs. 467.7 ms), lower in session 2 (484.4 vs. 549.0 ms) and again higher in session 3 (478.3 vs. 453.7 ms).

### Decision points

A repeated measures ANOVA with the between factors age group (young, old) and the within factors session (1–3) and movement direction (straight, turn) revealed main effects of age group [*F*_(1, 43)_ = 20.80, *p* < 0.001] and movement direction [*F*_(1, 43)_ = 23.52, *p* < 0.001], but no main effect of session [*F*_(2, 86)_ = 1.92, *p* = 0.15]. Specifically, younger participants responded faster than older participants (446 vs. 709 ms) and response times were shorter for straight movements than for turns (516 vs. 604 ms, see Figure 3C).

All two way interactions rendered significant results: age group × session [*F*_(2, 86)_ = 7.26, *p* = 0.001], age group × movement direction [*F*_(1, 43)_ = 4.75, *p* = 0.03] and session × movement direction [*F*_(2, 86)_ = 6.31, *p* = 0.003]. The age group × session interaction was driven by increasing response times over experimental sessions in the older participants group (from 651 ms in session 1 to 760 ms in session 3; *p* < 0.05, see Figure 3B). The age group × movement interaction is driven by a larger difference in response times between straight movements and turns in the older participant group (straight movements: 624 ms; turns: 751 ms) than in the younger participant group (straight movements: 412 ms; turns: 463 ms; see Supplementary Figure 1D). The session x movement direction interaction was driven by a greater difference in response times between turns and straight movements in session 2 as compared to session 1 or 3 (difference between turns and straight movements in session 1: 70 ms; session 2: 148 ms; session 3: 32 ms; see Supplementary Figure 1C).

### Is attentional engagement predictive for route learning?

To investigate how attentional engagement with the route learning task is associated with actual route learning performance we correlated differences between response times at decision points and non-decision points with participants' performance for decision points in the Landmark-Only test. For both, our young and our old participant group we found similar significant positive correlations [young participants: *r*_(21)_ = 0.54; *p* < 0.01; old participant group: *r*_(21)_ = 0.58; *p* < 0.01; see Supplementary Figure 2]. In other words, the greater the increase in response times to the auditory probe at decision points compared to non-decision points, the better the performance in the final test assessing route knowledge.

## Discussion

In this study we used an auditory-probe task to investigate the impact of the navigational relevance of spatial situations along a route through a virtual environment on attentional engagement. To investigate whether attentional engagement contributes to aging-related route-learning deficits we compared route learning performance and response times to the auditory probes between a young and an older participant group. As expected, we found reduced route learning performance in the older adult group as compared to the younger group. Our older participants were slower than the young participants to respond to the auditory probes during route learning and both the young and older participants' response times to the auditory probe were longer at decision points than at non-decision points. Finally, differences in response times to the auditory-probe task between decision points and non-decision points were associated with route learning performance.

Route learning performance was measured with two tests that both assessed landmark-direction associations. The Landmark-In-Context test was administered twice between the training sessions and used screenshots as stimuli. In line with earlier studies investigating route learning in virtual environments, these tests showed that older participants performed significantly worse than younger participants (Head and Isom, 2010; Wiener et al., 2012; Zhong and Moffat, 2016). Both the Landmark-in-Context and the Landmark-Only test showed that participants (young and old) performed best when presented with landmark objects from decision points that required moving straight on as opposed to making a turn. These results are consistent with the “when in doubts follow your nose strategy,” first described in the context of exploration behavior (Dalton, 2003) and later applied to route learning (Meilinger et al., 2012, 2014). This strategy states that the default movement direction at intersections is straight. Therefore, navigators do not have to explicitly encode straight movements, only turns, which would result in reduced memory load (see also Klippel et al., 2003).

The Landmark-Only test was administered only once after the last training session. In contrast to the Landmark-In-Context test, the landmark objects were presented in isolation in the Landmark-Only test which allowed us to study landmark-direction associations not only for decision points but also for non-decision points. Importantly, performance in the Landmark-Only test was better for landmark objects positioned at navigationally relevant locations (i.e., decision points) than for those positioned at non-decision points (i.e., turns or corridors). This suggests that participants paid particular attention to the navigationally relevant decision points, while fewer attentional resources were devoted to monitor and learn other parts of the route (Aginsky et al., 1997; Janzen and van Turennout, 2004; Janzen, 2006; Janzen and Weststeijn, 2007; Schinazi and Epstein, 2010).

The performance differences between decision points and non-decision points are reflected in the response times to the auditory probes which are thought to capture attentional engagement with the main task (Posner and Boies, 1971). Specifically, both participant groups responded faster to auditory probes at non-decision points as compared to decision points. This is consistent with our predictions and suggests that participants allocate more attentional resources to the route learning task when they approach navigationally relevant locations such as, intersections as compared to simple turns (Allen and Kirasic, 2003). Interestingly, this effect was primarily driven by an increase in response times at decision points when the route turned. It is important to note at this point that this effect cannot simply be explained by the onset of a rotational movement, potentially a salient stimulus capturing attention, as rotational movement happen at both decision as well as non-decision points. We argue that the increased response time rather reflects the additional attention and processing required to successfully encode the landmark direction association at decision points with changes in movement direction. Straight movements, in contrast, may not need to be represented if one assumes that the default movement direction is straight (see “When in doubt follow your nose strategy” discussed above; Meilinger et al., 2012, 2014).

Differences in response times to auditory probes presented at decisions points and non-decision points were associated with route learning success. In other words, the longer participants needed to respond to a probe at a decision as compared to a non-decision point, the better their performance in the final Landmark-Only test. This result demonstrates that the auditory probe procedure not only captures differences in attentional engagement between navigationally relevant and irrelevant situations, but that these differences are in fact predictive for learning performance and therefore tap into attentional processes that are crucial for successful navigation. These results are in line with earlier studies using auditory probe procedures in different cognitive domains (e.g., Lansman and Hunt, 1982).

Our older participants needed significantly longer to respond to the auditory probes during training than the younger participants. This is consistent with theories of general aging-related declines in information processing speed (Salthouse, 1996, 2000; Glisky, 2007). Contrary to our predictions, however, the data suggests that our older participants did effectively control attentional resources in order to engage with the route learning task when approaching navigationally relevant situations. If that was not the case, response times in the older participant group should not have differed—or differed substantially less—between decision and non-decision points as compared to the young participants. We did, however, find that the response times to the auditory probes presented at decision points increased in the older participant group across sessions, while they stayed the same in the younger group. Importantly, this effect was absent for non-decision points. These results suggest that our older participants took longer before they started directing attentional resources to the navigationally relevant decision points. This interpretation is in line with results from the Landmark-In-Context test which showed that our older participants did not exceed chance level performance in the first test session. Together with the fact that route learning performance was correlated with the magnitude of the difference in response times between DPs and non-DPs, these results suggest that directing attentional resources to the navigationally relevant situations is an important factor contributing to successful route learning, and that our older participants only did so efficiently in the second training session, i.e., it might take them longer to attend to the navigationally relevant information. In addition to more general associative learning deficits in older adults (Naveh-Benjamin et al., 2007, 2009), this could contribute to route learning differences between age groups and is consistent with earlier research suggesting that older adults regard salient landmarks rather than navigationally relevant situations as route critical elements (Lipman, 1991).

It should be noted at this point, that the virtual environment used in the current study was very simple. Moreover, instead of actively navigating through the environment, participants watched a pre-recorded video of the route during the experiment. Even though several studies have demonstrated very similar results when comparing route learning and navigation behavior in real and virtual environments (e.g., Cushman et al., 2008; van der Ham et al., 2015), and other studies have shown that route learning performance did not differ between active and passive route exploration (e.g., Cutmore et al., 2000; Gaunet et al., 2001), it is important to replicate the findings presented here in more realistic navigation scenarios.

While our approach allowed us to isolate the impact of navigational relevance on route learning, landmark objects and the geometry were the only environmental cues that could attract attention. More complex naturalistic environments, in contrast, will feature many environmental cues that are not navigationally relevant. Given that research in other cognitive domains has shown that older adults have more difficulties ignoring salient but task-irrelevant cues (Schmitz et al., 2010; Tsvetanov et al., 2013), one might expect that the effects we presented here could even be emphasized when learning a route through a more naturalistic environments.

## Ethics statement

This study was carried out in accordance with the recommendations of the Research Ethics Code of Practice, Science, Technology Health Research Ethics Panel at Bournemouth University with written informed consent from all subjects. All subjects gave written informed consent in accordance with the Declaration of Helsinki. The protocol was approved by the Science, Technology Health Research Ethics Panel at Bournemouth University.

## Author contributions

Conception or design of the work: SH, JW, and TW. Data collection: SH and RG. Data analysis and interpretation: SH, RG, and JW. Drafting the article: SH, RG and JW Critical revision of the article SH, RG, JW, and TW. Final approval of the version to be published: SH, RG, JW, and TW.

### Conflict of interest statement

The authors declare that the research was conducted in the absence of any commercial or financial relationships that could be construed as a potential conflict of interest.

## Supplementary material

All data created during this research are openly available from the UK Data Service archive at http://reshare.ukdataservice.ac.uk/852534/.
