# Supplementary material for: The Effects of Attentional Engagement on Route Learning Performance in a Virtual Environment: An Aging Study
Source: Front Aging Neurosci. 2017 Jul 20;9:235. doi: 10.3389/fnagi.2017.00235 (PMC5517407; doi:10.3389/fnagi.2017.00235)
Supplement: Supplementary file 1 [file DataSheet1.PDF]

Supplementary Material

The effects of attentional engagement on route learning performance in a virtual environment: an aging study

Steffen Hartmeyer[1], Ramona Grzeschik[1], Thomas Wolbers[2], Jan M. Wiener[1]

[1]Bournemouth University, Faculty of Science & Technology, Department of Psychology & Dementia Institute

[2]German Centre for Neurodegenerative Diseases, Magdeburg, Germany

*** Correspondence:** Ramona Grzeschik: rgrzeschik@bournemouth.ac.uk

1. Video of the route

The compressed video "Experiment.avi" shows the route through the virtual environment without the auditory probe.

2. Supplementary Figures

Suppl. Figure 1 shows the significant interactions revealed for the auditory probe task.

Suppl. Figure 2 shows the correlation between reaction time in the auditory probe task and performance in the Landmark-Only test.

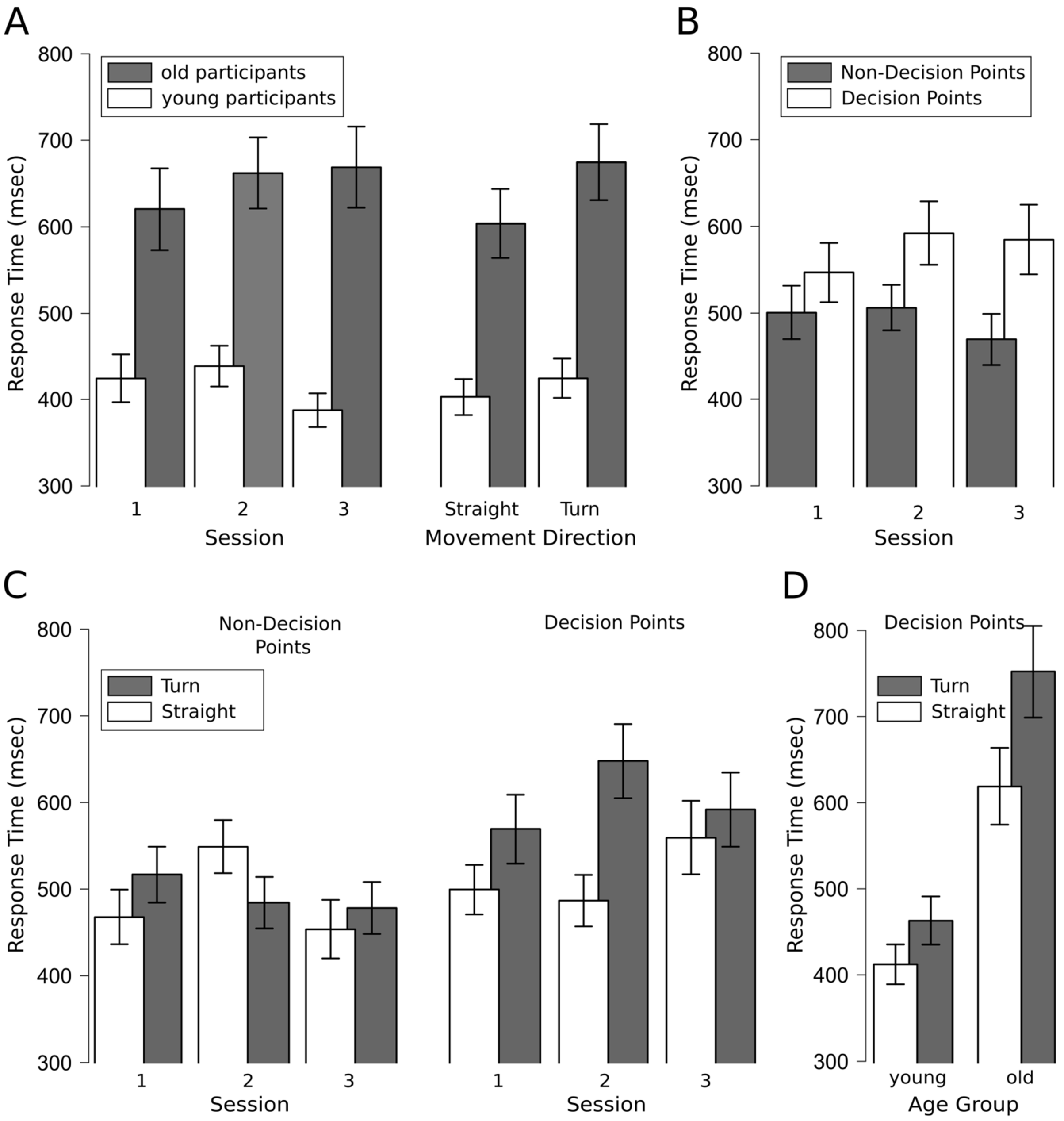

Supplementary Figure 1: Significant interactions of the response times revealed for the auditory-probe task. A (left): age group x session ($p < 0.01$); A (right): age group x movement direction ($p = 0.02$); B: decision point x session ($p = 0.03$); C: decision x movement x session ($p < 0.001$) with the single interactions session x movement at non-decision points ($p = 0.003$; left) and at decision points ($p = 0.003$; right); D: interaction age group x movement direction at decision points only ($p = 0.03$).

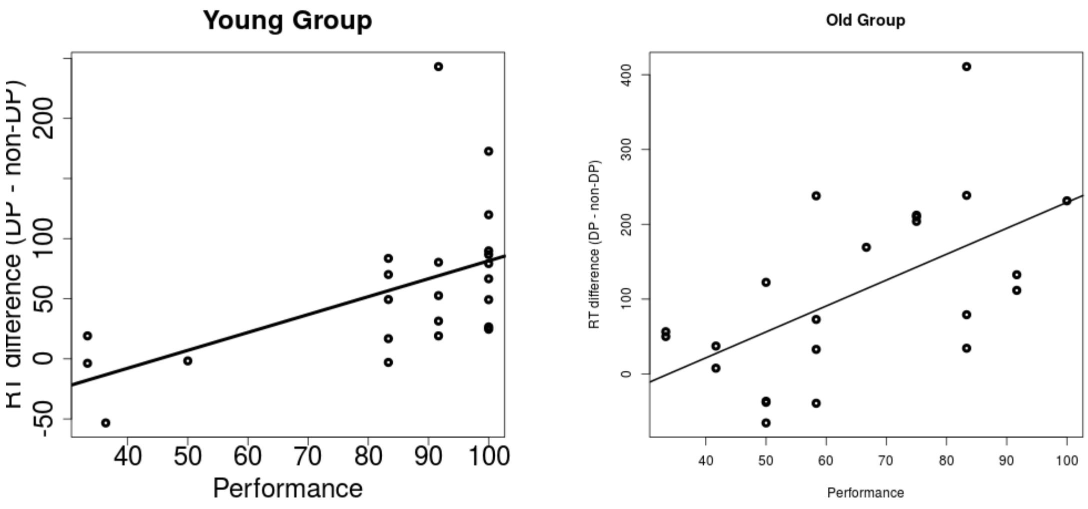

Supplementary Figure 2: Correlation between the difference of response times at decision points and non-decision points with participants' performance for decision points in the final Landmark-Only test. For both age groups, performance and RT difference correlated significantly (young participants: $r(21)=0.54$; $p<0.01$, left panel; old participant group: $r(21) = 0.58$; $p<0.01$, right panel).
